# Intravenous thrombolysis with 0.65 mg/kg r-tPA may be optimal for Chinese mild-to-moderate stroke

**DOI:** 10.3389/fneur.2022.989907

**Published:** 2022-09-20

**Authors:** Yu Cui, Zhi-Guo Yao, Hui-Sheng Chen

**Affiliations:** Department of Neurology, General Hospital of Northern Theatre Command, Shenyang, China

**Keywords:** acute ischemic stroke, intravenous thrombolysis, optimal dose, recombinant tissue plasminogen activator, China

## Abstract

**Background:**

Intravenous recombinant tissue plasminogen activator (r-tPA) with 0.9 mg/kg is the standard treatment for acute ischemic stroke, but it remains unclear whether it is optimal for all patients. We aimed to determine the optimal dose of r-tPA for Chinese stroke based on the data from the INTRECIS study.

**Methods:**

From the INTRECIS cohort, patients receiving intravenous r-tPA within 4.5 h of onset were included. According to r-tPA dose, patients were assigned into seven groups (from 0.60 to 0.90 mg/kg). The primary outcomes were the proportion of excellent functional outcomes and symptomatic intracranial hemorrhage.

**Results:**

Overall, 2,666 patients were included: 156 in 0.60 mg/kg group, 117 in 0.65 mg/kg group, 127 in 0.70 mg/kg group, 188 in 0.75 mg/kg group, 154 in 0.80 mg/kg group, 359 in 0.85 mg/kg group, and 1,565 in 0.90 mg/kg group. After adjustment for baseline characteristics, only 0.65 mg/kg group had significantly higher proportion of excellent functional outcome than 0.90 mg/kg group (79.5 vs. 71.4%, odds ratio = 1.833, 95% CI = 1.006–3.341, adjusted *p* = 0.048). The subgroup analysis showed no evidence of differences in the odds of having a primary outcome between the two groups by age, admission NIHSS, onset to thrombolysis time, and TOAST classification. There was no significant difference in symptomatic intracranial hemorrhage between groups.

**Conclusion:**

Our study presented the first evidence that intravenous thrombolysis with 0.65 mg/kg r-tPA may be optimal for Chinese mild-to-moderate stroke.

**Registration:**

https://www.clinicaltrials.gov, identifier: NCT 02854592.

## Introduction

Intravenous recombinant tissue plasminogen activator (r-tPA) with 0.9 mg/kg is an effective and guideline-recommended treatment for acute ischemic stroke (AIS) worldwide (1–3). Given the possible dose-related difference in effectiveness and safety in a different population, the optimal dose of r-tPA has been a research focus.

The Japan alteplase clinical trial firstly showed the possibly comparable benefits of 0.6 mg/kg r-tPA to the 0.9 mg/kg (4). However, subsequent several studies in Asian regions showed inconsistent results of low-dose versus standard-dose in AIS (5–10). The enhanced control of hypertension and thrombolysis stroke study did not demonstrate the non-inferior effect of low-dose r-tPA to the standard-dose but indicated fewer symptomatic intracranial hemorrhage (sICH) with low-dose r-tPA (11). Given that previous studies mostly focused on the “rigid” dose of r-tPA (0.6 or 0.9 mg/kg) in AIS, the optimal dose of r-tPA deserves further investigation, especially in a large-sample cohort with multiple doses used.

INtravenous Thrombolysis REgistry for Chinese Ischaemic Stroke (INTRECIS) within 4.5 h of onset is a “real world,” national, and multi-center registry study in China, which included patients with AIS treated with multiple doses of r-tPA (12). In the present study, we compared the effectiveness and safety outcomes of multi-dose r-tPA in the INTRECIS cohort, aiming to determine the optimal dose of r-tPA for AIS in China.

## Methods

### Study design

From the INTRECIS cohort, all the patients receiving intravenous r-tPA (Boehringer Ingelheim Pharma GmbH & Co) within 4.5 h were enrolled in the present study. The detailed inclusion/exclusion criteria and study design have been reported (12). In brief, consecutive adult patients (age ≥18 years) with brain imaging confirmed AIS who were previously well [modified Rankin Scale (mRS) scores 0 or 1] and eligible for treatment with intravenous r-tPA within 4.5 h of a definite time of onset of symptoms were enrolled, including those with large vessel occlusion and undergoing endovascular revascularization therapy. All the demographic, clinical, and functional assessment data were obtained at admission and follow-up.

Participating patients with AIS received different doses of r-tPA (0.6–0.9 mg/kg), according to their age and neurological severity. For example, a higher dose was generally chosen for younger patients with greater neurological severity on the National Institutes of Health Stroke Scale (NIHSS), whereas lower doses were used for older patients with lower NIHSS scores (12). According to r-tPA dose used, the patients were divided into seven groups: 0.60 mg/kg (range from 0.60 to 0.625) group, 0.65 mg/kg (range from 0.625 to 0.675) group, 0.70 mg/kg (range from 0.675 to 0.725) group, 0.75 mg/kg (range from 0.725 to 0.775) group, 0.80 mg/kg (range from 0.775 to 0.825) group, 0.85 mg/kg (range from 0.825 to 0.875) group, and 0.90 mg/kg (range from 0.875 to 0.90) group. As the standard dose of r-tPA, the 0.90 mg/kg group was compared with the other six groups, respectively.

### Outcomes measurements

The primary effectiveness and safety outcomes were excellent functional outcomes and sICH, respectively. The excellent functional outcome was defined as scores of 0–1 on the mRS, which was assessed by face-to-face or a telephone interview at 90 days. The sICH was defined as an increase of ≥4 on scores on the National Institutes of Health Stroke Scale (NIHSS) caused by intracranial hemorrhage within 36 h, with all the clinician-reported details centrally adjudicated (13). All the patients with neurological deterioration received computerized tomography or magnetic resonance imaging to identify the occurrence of intracranial hemorrhage. The secondary outcomes included the proportion of patients with mRS scores of 0–2, mRS scores distribution, other bleeding events, recurrent stroke, and all-cause death at 90 days.

### Statistical analysis

First, we conducted descriptive analyses for baseline characteristics in groups. Continuous variables with normal and abnormal distribution were described as means (SD) and median (interquartile range), respectively. Continuous variables included age, systolic blood pressure, diastolic blood pressure, symptom onset to thrombolysis time, door to needle time, and NIHSS score. Categorical variables were described as numbers (proportions). Categorical variables included gender, current smoker, current drinker, hypertension, coronary heart disease, history of stroke, diabetes mellitus, atrial fibrillation, and Trial of Org 10172 in Acute stroke treatment (TOAST) classification (14).

Second, we conducted descriptive analyses for primary and secondary outcomes in groups. To identify the optimal dose of r-tPA, we conducted binary logistic regression analyses of excellent functional outcome by comparing other dose of r-tPA groups with the standard dose of r-tPA (0.90 mg/kg) group, and the sensitivity analysis. In the binary logistic regression analyses, the group was set as an independent variable, of which the 0.90 mg/kg group was defined as the reference category, and excellent functional outcome was set as the dependent variable.

Third, the identified optimal dose of the r-tPA group, which showed better excellent functional outcome with statistical significance of difference than the standard dose of the r-tPA group, was further compared with the standard dose of the r-tPA group in sICH, and secondary outcomes of the proportion of patients with mRS scores 0–2, other bleeding events, recurrent stroke, and all-cause death at 90 days through binary logistic regression analyses. The mRS scores distribution at 90 days was compared through shift analysis using ordinal logistic regression analysis. We also did sensitivity analyses.

In the present study, sensitivity analyses were performed by adjusting for baseline characteristics (age, gender, current smoker, current drinker, hypertension, coronary heart disease, history of stroke, diabetes mellitus, atrial fibrillation, systolic blood pressure, diastolic blood pressure, symptom onset to thrombolysis time, door to needle time, NIHSS score, and TOAST classification).

Fourth, to identify who will benefit from the optimal dose, we also assessed the consistency of excellent functional outcomes across four prespecified subgroups through tests for interaction in binary logistic regression analysis. [age (<65 or ≥65 years), gender (male or female), symptom onset to thrombolysis time ( ≤ 180 or >180 min), admission NIHSS score (0–5, 6–10 or >10), and TOAST classification (large-artery atherosclerosis, cardioembolic, small-artery occlusion, other determined cause, and undetermined cause)].

In the present study, results were reported with odds ratio (OR) and 95% CI. In the relevant analytic tests, differences were considered statistically significant with a *p* < 0.05. The statistical software SPSS version 23.0 (IBM, NY, USA) was used for the outcomes and graphs in the analysis.

## Results

**Figure 1:**
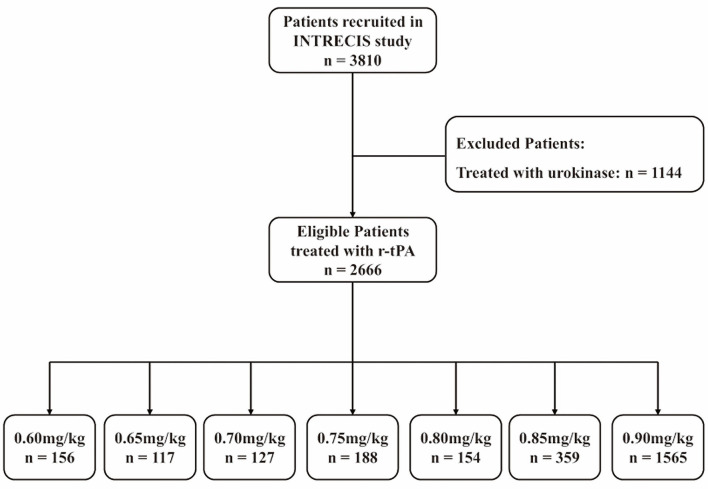
Flow diagram. r-tPA, recombinant tissue plasminogen activator; INTRECIS, INtravenous Thrombolysis REgistry for Chinese Ischaemic Stroke within 4.5 h of onset.

**Figure 2:**
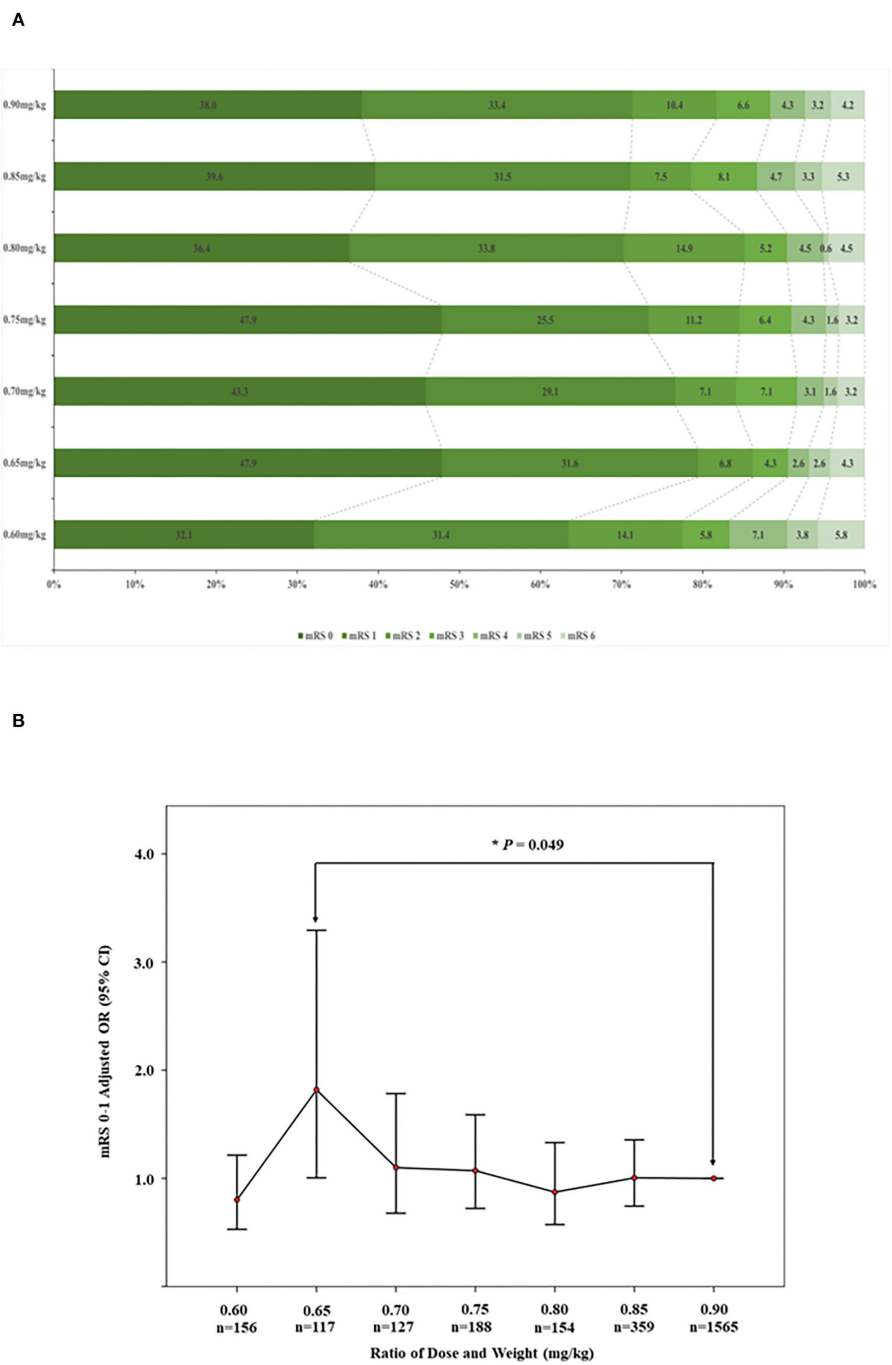
**(A)** Comparison of mRS scores at 90 days by the dose of r-tPA. mRS, modified Rankin scale. **(B)** Adjusted comparing in proportion of 90-day modified Rankin Scale scores 0–1 in 0.65 vs. 0.9 mg/kg groups. OR, odds ratio; CI, confidence interval.

In the INTRECIS cohort, 2,666 patients receiving intravenous r-tPA within 4.5 h were included in the present study: 156 patients in 0.60 mg/kg group, 117 patients in 0.65 mg/kg group, 127 patients in 0.70 mg/kg group, 188 patients in 0.75 mg/kg group, 154 patients in 0.80 mg/kg group, 359 patients in 0.85 mg/kg group, and 1,565 patients in 0.90 mg/kg group (Figure 1). Baseline characteristics of patients in each group were shown in Table 1. Table 2 showed the effectiveness and safety outcomes in groups. The highest proportion of mRS 0–1 (79.5%) and mRS 0–2 (86.3%) was found in the 0.65 mg/kg group (Table 2; Figure 2A).

**Table 1:** Baseline characteristics of patients in multi-dose r-tPA groups.

**Variable**	**0.60 mg/kg** **(*N* = 156)**	**0.65 mg/kg** **(*N* = 117)**	**0.70 mg/kg** **(*N* = 127)**	**0.75 mg/kg** **(*N* = 188)**	**0.80 mg/kg** **(*N* = 154)**	**0.85 mg/kg** **(*N* = 359)**	**0.90 mg/kg** **(*N* = 1,565)**	***P*** **-value**
Age, years	69 (60–79)	63 (54–75)	64 (55–70)	65 (58–74)	65 (59–74)	63 (55–70)	64 (56–72)	<0.001
Gender, male	104 (66.7)	101 (86.3)	102 (80.3)	135 (71.8)	96 (62.3)	242 (67.4)	1,047 (66.9)	<0.001
Current smoker	59 (37.6)	55 (47.0)	56 (44.1)	68 (36.2)	49 (31.8)	128 (35.7)	591 (37.8)	0.142
Current drinker	37 (23.7)	31 (26.5)	29 (22.8)	41 (21.8)	36 (23.4)	82 (22.8)	351 (22.4)	0.975
Hypertension	80/154 (51.9)	55/114 (48.2)	62/124 (50.0)	96/185 (52.7)	83/151 (55.0)	180/347 (51.9)	879/1,524 (57.7)	0.109
Coronary heart disease	35/150 (23.3)	15/113 (13.3)	18/122 (14.8)	30/183 (16.4)	22/148 (14.9)	51/342 (14.9)	223/1,491 (15.0)	0.231
History of stroke	46/153 (30.1)	30/114 (26.3)	31/125 (24.8)	39/182 (21.4)	32/152 (21.1)	75/341 (22.0)	279/1,506 (18.5)	0.012
Diabetes mellitus	37/154 (24.0)	29/116 (25.0)	20/126 (15.9)	37/184 (20.1)	19/150 (12.7)	63/346 (18.2)	302/1,524 (19.8)	0.114
Atrial fibrillation	19/148 (12.8)	7/116 (6.0)	10/124 (8.1)	15/181 (8.3)	13/147 (8.8)	33/345 (9.6)	157/1,486 (10.6)	0.508
Systolic blood pressure, mmHg	148.2 (23.4)	150.3 (22.2)	149.2 (26.2)	146.7 (21.4)	153.9 (21.3)	151.2 (22.4)	151.9 (22.8)	0.022
Diastolic blood pressure, mmHg	85.2 (14.2)	88.4 (12.7)	89.8 (15.7)	85.1 (12.7)	89.1 (13.7)	88.6 (12.5)	89.0 (13.7)	<0.001
Symptom onset to thrombolysis time, min	167 (120–210)	180 (140–224)	165 (123–205)	170 (136–211)	165 (120–206)	169 (129–207)	170 (128–210)	0.227
Door to needle time, min	60 (40–85)	60 (37–87)	65 (42–101)	60 (38–90)	67 (42–99)	58 (38–85)	56 (35–85)	0.578
NIHSS score	5 (3–12)	5 (3–8)	5 (3–10)	5 (3–10)	6 (3–10)	6 (3–11)	6 (3–11)	0.097
**TOAST classification**								
Large-artery atherosclerosis	75/155 (48.4)	63/114 (55.3)	61/122 (50.0)	97/188 (51.6)	75/153 (49.0)	178/355 (50.1)	748/1,540 (48.6)	0.001
Cardioembolism	28/155 (18.1)	5/114 (4.4)	12/122 (9.8)	16/188 (8.5)	18/153 (11.8)	27/355 (7.6)	198/1,540 (12.9)	
Small-artery occlusion	37/155 (23.9)	43/114 (37.7)	37/122 (30.3)	60/188 (31.9)	53/153 (34.6)	127/355 (35.8)	457/1,540 (29.7)	
Other determined cause	1/155 (0.6)	1/114 (0.9)	7/122 (5.7)	6/188 (3.2)	4/153 (2.6)	6/355 (1.7)	38/1,540 (2.5)	
Undetermined cause	14/155 (9.0)	2/114 (1.8)	5/122 (4.1)	9/188 (4.8)	3/153 (2.0)	17/355 (4.8)	99/1,540 (6.4)	

Data are n/N (%), mean (standard deviation) or median (interquartile range).

r-tPA, recombinant tissue plasminogen activator; NIHSS, National Institutes of Health Stroke Scale; TOAST, Trial of Org 10172 in Acute Stroke Treatment.

P-value indicates a comparison between groups.

**Table 2:** Effectiveness and safety outcomes in multi-dose r-tPA groups.

**Outcome**	**0.60 mg/kg** **(*N* = 156)**	**0.65 mg/kg** **(*N* = 117)**	**0.70 mg/kg** **(*N* = 127)**	**0.75 mg/kg** **(*N* = 188)**	**0.80 mg/kg** **(*N* = 154)**	**0.85 mg/kg** **(*N* = 359)**	**0.90 mg/kg** **(*N* = 1,565)**	***P*** **-value**
mRS 0–1 at 90 days	99 (63.5)	93 (79.5)	92 (72.4)	138 (73.4)	108 (70.1)	255 (71.0)	1,117 (71.4)	0.166
mRS 0–2 at 90 days	121 (77.6)	101 (86.3)	101 (79.5)	159 (84.6)	131 (85.1)	282 (78.6)	1,279 (81.7)	0.214
**mRS distribution at 90 days**								
0	50 (32.1)	56 (47.9)	55 (43.3)	90 (47.9)	56 (36.4)	142 (39.6)	594 (38.0)	0.175
1	49 (31.4)	37 (31.6)	37 (29.1)	48 (25.5)	52 (33.8)	113 (31.5)	523 (33.4)	
2	22 (14.1)	8 (6.8)	9 (7.1)	21 (11.2)	23 (14.9)	27 (7.5)	162 (10.4)	
3	9 (5.8)	5 (4.3)	9 (7.1)	12 (6.4)	8 (5.2)	29 (8.1)	103 (6.6)	
4	11 (7.1)	3 (2.6)	4 (3.1)	8 (4.3)	7 (4.5)	17 (4.7)	68 (4.3)	
5	6 (3.8)	3 (2.6)	2 (1.6)	3 (1.6)	1 (0.6)	12 (3.3)	50 (3.2)	
6	9 (5.8)	5 (4.3)	11 (8.7)	6 (3.2)	7 (4.5)	19 (5.3)	65 (4.2)	
sICH at 36 h	3 (1.9)	0 (0.0)	2 (1.6)	2 (1.1)	2 (1.3)	4 (1.1)	17 (1.1)	0.866
Bleeding events at 90 days	3 (1.9)	0 (0.0)	2 (1.6)	1 (0.5)	4 (2.6)	5 (1.4)	15 (1.0)	0.352
Recurrent stroke at 90 days	2 (1.3)	1 (0.9)	2 (1.6)	2 (1.1)	2 (1.3)	6 (1.7)	21 (1.3)	0.995
Death in all-cause at 90 days	9 (5.8)	5 (4.3)	11 (8.7)	6 (3.2)	7 (4.5)	19 (5.3)	65 (4.2)	0.296

Data are n (%).

r-tPA, recombinant tissue plasminogen activator; mRS, modified Rankin Scale; sICH, symptomatic intracranial hemorrhage.

P-value indicates a comparison between groups.

**Figure 3:**
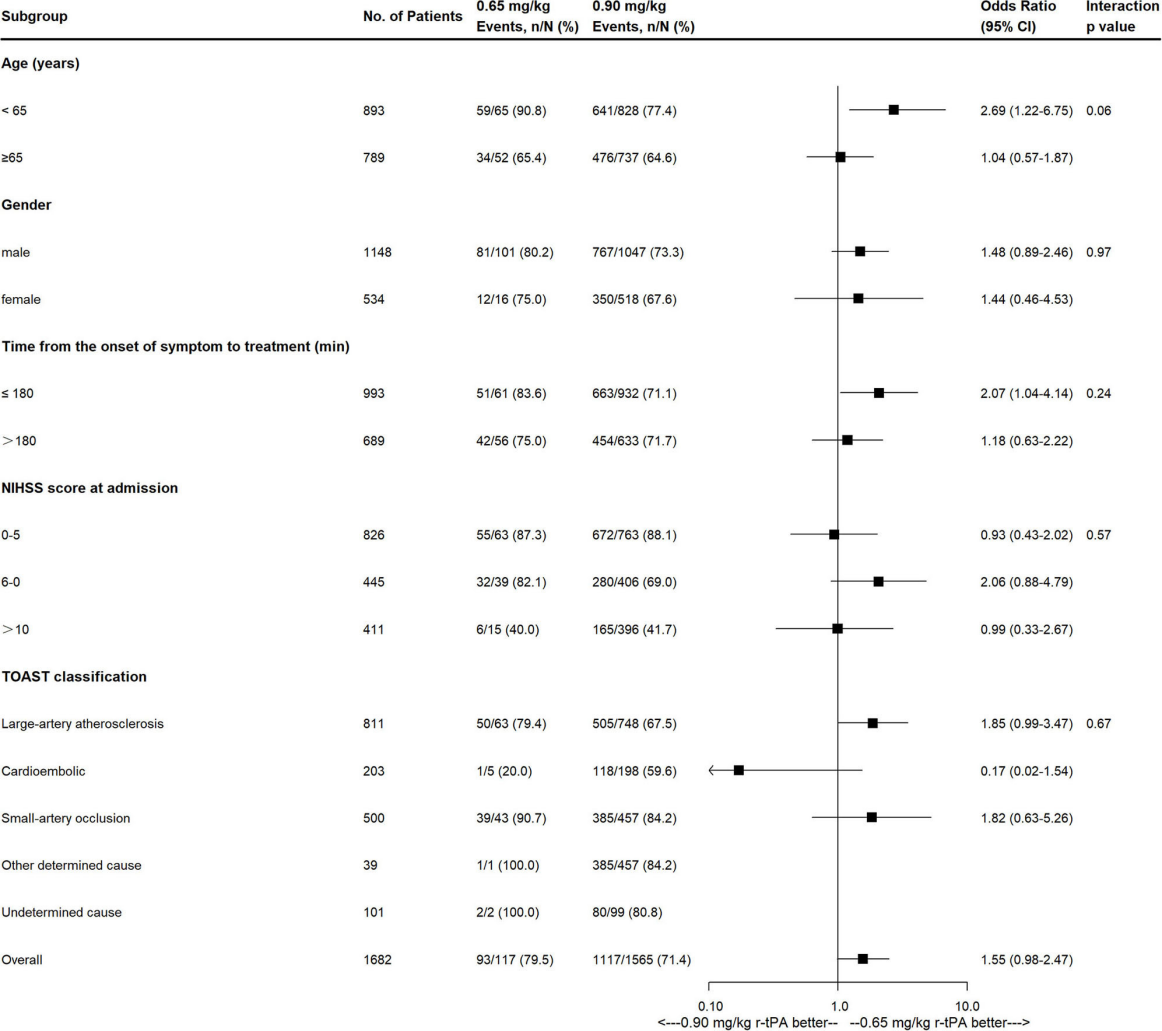
Effects of 0.65 mg/kg as compared with 0.9 mg/kg on excellent functional outcome according to prespecified subgroups. NIHSS, National Institutes of Health Stroke Scale; TOAST, Trial of Org 10172 in Acute Stroke Treatment classification.

Compared with the 0.90 mg/kg group, only 0.65 mg/kg group was found to have higher proportion of excellent functional outcome with significant difference after adjusting for baseline characteristics (79.5 vs. 71.4%, OR = 1.833, 95% CI = 1.006–3.341, adjusted *p* = 0.048; Figure 2B). In addition, mRS distribution at 90 days showed significant different in 0.65 vs. 0.90 mg/kg group before (OR = 1.493, 95% CI = 1.051–2.119, *p* = 0.025) and after adjusting for baseline characteristics (OR = 1.672, 95% CI = 1.125–2.487, adjusted *p* = 0.011), while other outcomes showed no significant difference (Table 3). Subgroup analysis showed no significant differences in the excellent functional outcome between the 0.65 and 0.90 mg/kg group by age, gender, NIHSS score at admission, onset to thrombolysis time, and TOAST classification (Figure 3).

**Table 3:** Effectiveness and safety outcomes between 0.65 and 0.90 mg/kg r-tPA groups.

**Outcome**	**0.65 mg/kg** **(*N* = 117)**	**0.90 mg/kg** **(*N* = 1,565)**	**Unadjusted**		**Adjusted**	
			**OR (95% CI)**	***P*** **-value**	**OR (95% CI)**	***P*** **-value**
mRS 0–1 at 90 days	93 (79.5)	1,117 (71.4)	1.554 (0.979–2.467)	0.061	1.833 (1.006–3.341)	0.048
mRS 0–2 at 90 days	101 (86.3)	1,279 (81.7)	1.412 (0.820–2.429)	0.213	1.350 (0.664–2.744)	0.407
mRS distribution at 90 days			1.493 (1.051–2.119)	0.025	1.672 (1.125–2.487)	0.011
sICH at 36 h	0 (0.0)	17 (1.1)		0.996		0.996
Bleeding events at 90 days	0 (0.0)	15 (1.0)		0.996		0.996
Recurrent stroke at 90 days	1 (0.9)	21 (1.3)	1.578 (0.210–11.833)	0.657	1.605 (0.201–12.836)	0.655
Death in all-cause at 90 days	5 (4.3)	65 (4.2)	1.030 (0.407–2.610)	0.950	0.398 (0.049–3.230)	0.389

Data are n (%).

r-tPA, recombinant tissue plasminogen activator; mRS, modified Rankin Scale; OR, odds ratio; CI, confidence interval; sICH, symptomatic intracranial hemorrhage.

Furthermore, we performed univariate and multivariate analysis for the predictors of excellent functional outcome and sICH in all patients, the results of which were respectively shown in Tables 4, 5.

**Table 4:** Univariate and multivariate logistic regression analysis to predict excellent functional outcome (OR and 95% CI).

**Variable**	**Univariate analysis**			**Multivariate analysis**		
	**OR**	**95% CI**	***P*** **-value**	**OR**	**95% CI**	***P*** **-value**
Age, years	0.967	0.960–0.975	<0.001	0.979	0.969–0.989	<0.001
Gender, male	0.801	0.670–0.957	0.014	0.951	0.743–1.216	0.688
Current smoker	0.908	0.763–1.081	0.278	1.085	0.841–1.399	0.530
Current drinker	0.714	0.579–0.880	0.002	0.752	0.563–1.003	0.052
Hypertension	0.868	0.731–1.030	0.106	0.986	0.795–1.223	0.899
Coronary heart disease	0.821	0.651–1.035	0.095	1.047	0.785–1.396	0.757
History of stroke	0.802	0.653–0.986	0.036	0.871	0.682–1.114	0.272
Diabetes mellitus	0.919	0.743–1.136	0.273	1.028	0.793–1.331	0.837
Atrial fibrillation	0.495	0.380–0.646	<0.001	1.040	0.738–1.465	0.825
Systolic blood pressure, mmHg	0.995	0.991–0.999	0.006	0.997	0.992–1.003	0.387
Diastolic blood pressure, mmHg	0.996	0.990–1.002	0.227	0.997	0.988–1.007	0.587
Symptom onset to thrombolysis time, min	1.000	0.998–1.001	0.943	0.999	0.997–1.001	0.453
Door to needle time, min	0.999	0.998–1.001	0.383	1.000	0.998–1.002	0.872
NIHSS score	0.858	0.844–0.873	<0.001	0.871	0.855–0.887	<0.001
TOAST classification	1.325	1.224–1.434	<0.001	1.166	1.065–1.276	0.001

OR, odds ratio; CI, confidence interval; NIHSS, National Institutes of Health Stroke Scale; TOAST, Trial of Org 10172 in Acute Stroke Treatment.

**Table 5:** Univariate and multivariate logistic regression analysis to predict sICH (OR and 95% CI).

**Variable**	**Univariate analysis**			**Multivariate analysis**		
	**OR**	**95% CI**	***P*** **-value**	**OR**	**95% CI**	***P*** **-value**
Age, years	1.007	0.976–1.039	0.679	0.981	0.938–1.026	0.408
Gender, male	0.432	0.165–1.133	0.088	0.325	0.083–1.276	0.107
Current smoker	0.790	0.382–1.634	0.526	1.359	0.454–4.065	0.583
Current drinker	0.505	0.239–1.066	0.073	0.374	0.123–1.138	0.083
Hypertension	0.525	0.239–1.150	0.107	1.883	0.681–5.203	0.222
Coronary heart disease	0.429	0.187–0.987	0.047	2.103	0.714–6.195	0.178
History of stroke	1.254	0.476–3.303	0.647	0.635	0.202–1.994	0.437
Diabetes mellitus	0.632	0.278–1.436	0.273	0.980	0.316–3.040	0.973
Atrial fibrillation	0.271	0.118–0.621	0.002	3.698	1.119–12.222	0.032
Systolic blood pressure, mmHg	1.013	0.998–1.028	0.092	1.019	0.995–1.044	0.128
Diastolic blood pressure, mmHg	1.015	0.990–1.041	0.233	0.984	0.945–1.025	0.445
Symptom onset to thrombolysis time, min	0.995	0.989–1.001	0.127	0.997	0.989–1.005	0.444
Door to needle time, min	0.998	0.991–1.006	0.655	0.999	0.991–1.008	0.844
NIHSS score	1.057	1.014–1.102	0.008	1.040	0.984–1.100	0.163
TOAST classification	0.683	0.451–1.034	0.072	0.652	0.397–1.070	0.091

sICH, symptomatic intracranial hemorrhage; OR, odds ratio; CI, confidence interval; NIHSS, National Institutes of Health Stroke Scale; TOAST, Trial of Org 10172 in Acute Stroke Treatment.

## Discussion

The present study investigated the effectiveness and safety of intravenous thrombolysis with multi-dose r-tPA for Chinese AIS in a prospective, national, multi-center, and large-sample cohort. Compared with 0.90 mg/kg for AIS, we first found that 0.65 mg/kg may be an optimal dose of r-tPA for mild to moderate stroke, which exhibited a higher proportion of excellent functional outcome and similar safety profile.

Given different races of populations receiving intravenous r-tPA and the side-effects of r-tPA (15–17), low-dose r-tPA for AIS has been investigated in Asian regions, however, the results were inconsistent. For example, Japan Alteplase Clinical Trial explored 0.6 mg/kg r-tPA and first showed similar benefits comparable with 0.9 mg/kg r-tPA in Japanese population (4), which was also found in Korean population (8). For Chinese population, several studies investigated the issue: (1) Taiwan Thrombolytic Therapy for Acute Ischemic Stroke study compared the efficacy and safety of 0.72 vs. 0.90 mg/kg r-tPA and found that 0.90 mg/kg r-tPA may not be optimal for treating aged Chinese patients due to the lower functional independence, higher sICH, and mortality (6); (2) Thrombolysis Implementation and Monitor of Acute Ischemic Stroke in China study suggested that the standard-dose r-tPA had more favorable outcome than low-doses (0.64 or 0.79 mg/kg) r-tPA (7); (3) Early efficacy and safety were found to be not significantly different among the low-dose (0.6, 0.7, and 0.8 mg/kg) and standard-dose groups (9); (4) A cluster data analysis showed the comparable efficacy at discharge and lower risk of sICH of low-dose r-tPA than the standard-dose in patients who had a moderate stroke (10). In addition, The Enhanced Control of Hypertension and Thrombolysis Stroke Study with 63% Asian patients did not demonstrate the non-inferior effect of 0.6–0.9 mg/kg r-tPA in Asians, but showed comparable effectiveness outcomes in some subgroups (11). Taken together, we found that: (1) most of these above studies mainly focused on the comparison between two “rigid” doses of r-tPA (low dose vs. standard dose); (2) two studies compared the efficacy and safety of multiple doses of r-tPA in Chinese AIS, but were limited by relatively small sample and wider dose interval of low-dose groups.

So far, the INTRECIS study enrolled the largest Chinese population receiving intravenous thrombolysis with multiple doses of r-tPA in a cohort. Different from previous studies (6, 7), seven groups with more refined r-tPA dose intervals (±0.025 mg/kg) were used in the present study. The results showed that the 0.65 mg/kg group had a significantly higher proportion of excellent functional outcome than the 0.90 mg/kg group with a similar safety profile among groups. Furthermore, subgroup analysis showed no evidence of differences in the odds of having a primary outcome between the two groups by age, gender, admission NIHSS, onset to thrombolysis time, and TOAST classification. Given the possible effect of age on the primary outcome (*P* interaction = 0.06), younger patients may benefit from the 0.65 mg/kg dose, which deserves further investigation. The proportion of excellent functional outcome in the 0.65 mg/kg group was higher than those in other studies with a specified dose of 0.6 mg/kg (79.5 vs. 32.4, 33.1, 46.8%) (5, 8, 11), which could possibly be due to the inclusion of patients with predominantly mild neurological deficits (NIHSS scores median: 5 vs. 15, 15, 8). For the safety profile, post-thrombolytic sICH was the most feared complication and was associated with poor outcomes in clinical practice (18). According to the definition of sICH in the ECASS-II (14), the rates of sICH in patients receiving intravenous r-tPA with 0.6 and 0.9 mg/kg were 3.5 and 4.6% in the previous studies, respectively (5, 19). In the present study, the rates of sICH in 0.65 mg/kg group and 0.90 mg/kg group were 0.0 and 1.1%, respectively. The lower rates could be explained by the inclusion of patients with mild neurological deficits (20). Our study showed a similar rate of sICH in the 0.65 mg/kg group and 0.90 mg/kg group, which was consistent with a recent meta-analysis (21).

The present study was a secondary analysis of the INTRECIS study (12), which was the largest-sample cohort that included multiple doses of intravenous r-tPA for Chinese AIS, however, several limitations still remained in the current analysis. First, as a secondary analysis of INTRECIS study, the lack of randomization and conduct was limited to Chinese consisted in the current analysis. Although we performed multivariable logistic regression with adjusting baseline characteristics, the confounding bias due to the nature of the registry study and unbalanced in the sample size of each dose group may be not fully weakened. Second, although refined dose division was used in the present study, a dose interval could not represent the specified dose actually. Last, the main characteristics of the population limited our conclusions to Chinese AIS with mild to moderate neurological deficits.

## Conclusion

For the first time, our study suggested that 0.65 mg/kg may be an optimal dose of intravenous r-tPA for Chinese acute mild to moderate ischemic stroke, which warranted to be confirmed by random clinical trials in the future.

## Data availability statement

The raw data supporting the conclusions of this article will be made available by the authors, without undue reservation.

## Ethics statement

The studies involving human participants were reviewed and approved by General Hospital of Northern Theater Command Ethics Committee. The patients/participants provided their written informed consent to participate in this study.

## Author contributions

H-SC conceived and designed the study. YC analyzed the data and wrote the first draft of the manuscript. Z-GY acquired the data. All authors contributed to the article and approved the submitted version.

## Funding

This study was funded by grants from the National Key R&D Program of China (2017YFC1308203).

## Conflict of interest

The authors declare that the research was conducted in the absence of any commercial or financial relationships that could be construed as a potential conflict of interest.

## Publisher's note

All claims expressed in this article are solely those of the authors and do not necessarily represent those of their affiliated organizations, or those of the publisher, the editors and the reviewers. Any product that may be evaluated in this article, or claim that may be made by its manufacturer, is not guaranteed or endorsed by the publisher.
